# Monoclonal antibodies constructed from COVID-19 convalescent memory B cells exhibit potent binding activity to MERS-CoV spike S2 subunit and other human coronaviruses

**DOI:** 10.3389/fimmu.2022.1056272

**Published:** 2022-12-22

**Authors:** Yuan Peng, Yongcheng Liu, Yabin Hu, Fangfang Chang, Qian Wu, Jing Yang, Jun Chen, Shishan Teng, Jian Zhang, Rongzhang He, Youchuan Wei, Mihnea Bostina, Tingrong Luo, Wenpei Liu, Xiaowang Qu, Yi-Ping Li

**Affiliations:** ^1^ College of Animal Sciences and Veterinary Medicine, Guangxi University, Nanning, Guangxi, China; ^2^ Translational Medicine Institute, The First People’s Hospital of Chenzhou, Hengyang Medical School, University of South China, Chenzhou, China; ^3^ Institute of Human Virology, Department of Pathogen Biology and Biosecurity, and Key Laboratory of Tropical Disease Control of Ministry of Education, Zhongshan School of Medicine, Sun Yat-sen University, Guangzhou, China; ^4^ School of Laboratory Medicine and Biotechnology, Southern Medical University, Guangzhou, China; ^5^ School of Public Health, Southern Medical University, Guangzhou, China; ^6^ Department of Microbiology and Immunology, University of Otago, Dunedin, New Zealand; ^7^ Department of Infectious Diseases, The Third Affiliated Hospital of Sun Yat-Sen University, Guangzhou, China

**Keywords:** COVID-19, convalescents, monoclonal antibody, coronavirus, middle eastern respiratory syndrome coronavirus, spike protein

## Abstract

**Introduction:**

The Middle East respiratory syndrome coronavirus (MERS-CoV) and the severe acute respiratory syndrome coronavirus 2 (SARS-CoV-2) are two highly contagious coronaviruses causing MERS and COVID-19, respectively, without an effective antiviral drug and a long-lasting vaccine. Approaches for diagnosis, therapeutics, prevention, etc., particularly for SARS-CoV-2 that is continually spreading and evolving, are urgently needed. Our previous study discovered that >60% of sera from convalescent COVID-19 individuals, but <8% from general population, showed binding activity against the MERS-CoV spike protein, indicating that SARS-CoV-2 infection boosted antibodies cross-reactive with MERS-CoV.

**Methods:**

To generate antibodies specific to both SARS-CoV-2 and MERS-CoV, here we screened 60 COVID-19 convalescent sera against MERS-CoV spike extracellular domain and S1 and S2 subunits. We constructed and characterized monoclonal antibodies (mAbs) from COVID-19 convalescent memory B cells and examined their binding and neutralizing activities against human coronaviruses.

**Results and Discussion:**

Of 60 convalescent serum samples, 34 showed binding activity against MERS-CoV S2, with endpoint titers positively correlated with the titers to SARS-CoV-2 S2. By sorting single memory B cells from COVID-19 convalescents, we constructed 38 mAbs and found that 11 mAbs showed binding activity with MERS-CoV S2, of which 9 mAbs showed potent cross-reactivity with all or a proportion of spike proteins of alphacoronaviruses (229E and NL63) and betacoronaviruses (SARS-CoV-1, SARS-CoV-2, OC43, and HKU1). Moreover, 5 mAbs also showed weak neutralization efficiency against MERS-CoV spike pseudovirus. Epitope analysis revealed that 3 and 8 mAbs bound to linear and conformational epitopes in MERS-CoV S2, respectively. In summary, we have constructed a panel of antibodies with broad-spectrum reactivity against all seven human coronaviruses, thus facilitating the development of diagnosis methods and vaccine design for multiple coronaviruses.

## 1 Introduction

Coronaviruses are enveloped, single-stranded positive-sense RNA viruses belonging to the genus *Coronavirus* of the family *Coronaviridae* in the order *Nidovirales*. Animal coronaviruses cause respiratory, enteric, and neurological system diseases in a wide range of wildlife and domestic animals (1, 2). Human coronaviruses (hCoVs) mainly infect the respiratory tract causing respiratory symptoms ranging from mild to lethal. However, due to the changing habitat of host animals and the high plasticity of the viral receptor, animal coronaviruses have spread across species and caused infections in humans (3).

To date, seven coronaviruses have proven to transmit from animals to humans, including three highly pathogenic coronaviruses: the severe acute respiratory syndrome coronavirus 1 (SARS-CoV-1), SARS-CoV-2, and Middle East respiratory syndrome coronavirus (MERS-CoV), and four mild coronaviruses: 229E and NL63 (alpha [α] coronavirus) and OC43 and HKU1 (beta [β] coronavirus). The intermediate hosts of MERS-CoV and SARS-CoV-2 are dromedary camels and bats, respectively (1). Since first reported in Saudi Arabia and Jordan in 2012, MERS cases have been reported in 27 countries with a infection fatality rate of ~35% (4). SARS-CoV-2 infection caused coronavirus disease 2019 (COVID-19), first identified in 2019, which has spread globally, resulted in ~600 million infections and approximately 6.5 million deaths recorded by October 2022 (5). The fatality of COVID-19 varies substantially within the range of 0.0%-1.6%, much lower than estimations made earlier in the pandemic (6). Age, gender, co-infection with other pathogens, initial diseases, etc. relate to the severity of COVID-19 symptoms (7, 8). Infection of mild hCoVs often leads to common cold symptoms, however, individuals with severe combined immunodeficiency (SCID) may develop serious clinical symptoms and even death (9). While resolving the pandemics and therapy are important, probing and diagnosis of existing or emerging coronaviruses with potential outbreak as well as identifying the origin and intermediate hosts of coronaviruses could significantly prevent a pandemic to occur or restrict the spreading. Therefore, approaches for early diagnosis, prevention of cross transmission, and treatment of infections by multiple coronaviruses are urgently needed.

Coronavirus spike (S) protein extracellular domain (ECD) ectodomain comprises two functional subunits, S1 and S2. The S1 domain is responsible for recognition and binding to the receptor, while S2 determines the membrane fusion for viral entry into the cell (10). Different receptors recognize the S protein and mediate cell entry, such as human aminopeptidase N (APN) for HCoV-229E, angiotensin-converting enzyme 2 (ACE2) for HCoV-NL63, SARS-CoV-1, and SARS-CoV-2, and dipeptidyl peptidase 4 (DPP4) for MERS-CoV (10–14). The spike protein was shown to have multiple epitopes essential for eliciting immune responses (15), thus spike is main target of antibodies generated prophylactic and therapeutic purposes. The S2 subunit is also a potential target for neutralizing antibodies interfering with the rearrangement of the S protein and the insertion of fusion peptide, and it can be a vaccine targeted to elicit cross-immunity (16, 17). Considering that S2 is more conserved than S1, the fusion site of S2 could be an ideal target for epitope-focused vaccine development raising broadly neutralizing antibodies (nAbs) against multiple coronaviruses (10, 18).

Recently, we demonstrated that a small proportion (<8%) of the general population elicited antibodies against MERS-CoV S protein, while most of sera from COVID-19 convalescent individuals (>60%) showed binding to MERS-CoV S protein, indicating that SARS-CoV-2 infection boosted antibody response with cross-reactivity against MERS-CoV (19). Other studies also showed that plasma IgG from COVID-19 convalescents reacted with MERS-CoV S protein (20), and the mAbs targeting S2 subunit blocked MERS-CoV infection and showed cross-reactivity with betacoronaviruses (21). A MERS-CoV S2-targeting antibody isolated from immunized mice had cross-reaction with S proteins of eight betacoronaviruses (22). In addition, blood samples from Sierra Leoneans exposed to seasonal coronavirus contained antibodies cross-reactive to both SARS-CoV-2 and MERS-CoV (23). These observations suggest that there is a possibility of isolating antibodies with broad-spectrum reactivity against multiple coronaviruses from COVID-19 convalescent individuals.

In this study, we demonstrated that the majority of serum antibodies from 60 COVID-19 convalescents reacted with MERS-CoV S2 subunit and neutralized MERS-CoV pseudovirus. We constructed and identified 11 mAbs specific to MERS-CoV S2 subunit from single memory B cells from COVID-19 convalescents, of which 9 mAbs had cross-reactivity with other seven hCoVs S proteins. This study provides a panel of antibodies with broad-spectrum reactivity, thus facilitating the development of accurate diagnosis for multiple coronavirus infections.

## 2 Materials and methods

### 2.1 Human subjects

The study protocol was approved by the Institutional Review Board of Shaoyang Central Hospital, Hunan Province, China (V.1.0, 203200301), and all subjects or their legal guardians who participated in this study provided written informed consents. All patients were identified as SARS-CoV-2 infected according to the Guidelines for the Diagnosis and Treatment of COVID-19 (v.5) published by the National Health Commission of China, combined with clinical symptoms and quantitative PCR by the local health authorities. We collected information from 60 patients discharged from Shaoyang Central Hospital from January 23, 2020 to March 2, 2020, and the peripheral blood mononuclear cells (PBMCs) were drawn from the convalescents on the 28^th^ day after discharge (equivalent to 44-52 days after COVID-19 symptom onset) and used for antibody construction. PBMCs and sera were isolated with lymphocyte separation solution and stored in liquid nitrogen and -80°C, respectively. For serum antibody binding assays, blood from the 2^nd^, 5^th^, 8^th^, and 12^th^ months after recovery were taken.

### 2.2 ELISA analysis of serum antibodies binding to MERS-CoV spike protein

To determine the binding of serum antibody to MERS-CoV S protein, indirect ELISA was performed using spike (S) and subunits S1 and S2 were used as coating antigens. Briefly, 96-well plates were coated with MERS-CoV S, S1, and S2 (200 ng/well; S ECD, 40069-V08B; S1, 40069-V08H; S2, 40070-V08B; Sino Biological, China) in PBS at 4°C overnight. The plates were washed five times with PBS-T (0.05% Tween-20 in PBS) and then blocked with blocking buffer (2% FBS and 2% BSA in PBS-T) at room temperature for 2 h. The serum (1:1000 dilution, 100 μl/well) was added to the well and incubated at 37°C for 1 h, followed by addition of HRP-conjugated goat anti-human IgG (1:5000 dilution, 100 μl/well; D110150-0100, Sangon Biotech, China) and incubation at 37°C for 1 h. After washing five times with PBS-T, 3,3′,5,5′-tetramethylbenzidine (TMB, 100μl/well; 34029, Thermo Fisher Scientific, USA) was added and incubated at room temperature for 5 min. The reaction was stopped with 2M H_2_SO_4_. The absorbance (OD_450_ nm) was measured using a microplate reader (Thermo Fisher Scientific). The ratio of OD_450_ nm for each sample relative to the negative control was calculated and the value of positive/negative >3 was defined as a positive reaction.

To determine the endpoint titer of the serum antibody, 96-well plates were coated with 200 ng/well of MERS-CoV S2 at room temperature for 2 h. After washing five times with PBS-T, the wells were incubated with blocking buffer at 4°C overnight. A 4-fold serial dilution of serum (starting from 1:800) was added to the 96-well plate and incubated at 37°C for 1 h. The 96-well plate was washed five times and detected for antibody binding to MERS-CoV S2 using a microplate reader (Thermo Fisher Scientific), as described above. All experiments were performed using human healthy control (HC) sera as negative controls.

### 2.3 Avidity assay of serum antibodies

The avidity of serum IgG antibody to MERS-CoV-2 S2 was measured using a modified two-step method. In the first step, the serum dilutions were optimized to obtain OD_450_ nm values in the range of 0.5-1.5, which ensures a linear measurement of antibody avidity. The second step was the ELISA procedure described above, but with or without, as required, treating the plates with 1 M sodium thiocyanate (NaSCN) for 15 min after 1 h of antibody incubation (100 μl/well). The avidity index of the antibody was calculated by equation of OD NaSCN _1 M_/OD NaSCN _0 M_ × 100%.

### 2.4 Neutralization assay of serum antibodies

The neutralizing activity of the serum was determined by a reduction in luciferase expression after infection of ACE2-expressing 293T cells (ACE2-293T), developed in our lab, by the spike pseudovirus as described in a previous neutralization assay with SARS-CoV-2 pseudovirus (19). MERS-CoV-2 pseudovirus was cultured at 37°C with serial dilutions of serum samples for 1 h (dilutions, 1:30, 1:90, 1:270, 1:810, 1:2430, and 1:7290). The reaction mix was incubated with fresh ACE2-293T cells at 5% CO_2_ and 37°C for 24 h, and then the cells were lysed using 1× luciferase cell culture lysis buffer (E1510, Promega, USA), and luciferase activity was assessed using a Luciferase Assay System (E1510, Promega). The control wells containing only virus or cells were included in six replicates in parallel. Background relative light unit (RLU) values (cells only) were subtracted from each assay. Healthy control sera were used as negative controls, and guinea pig serum immunized with MERS-CoV spike protein were used as positive controls. ID_50_ was defined as a serum dilution with a 50% reduction in RLU values compared to the RLU of control solution wells (virus + cells). The cut-off value was defined as ID_50_ = 40, and ID_50_>40 was considered a neutralization effect. Neutralization titers were log_10_-transformed.

### 2.5 Neutralization of pseudovirus with mAbs

The ACE2-293T cells were plated in a 96-well plate (2×10^4^ cells/well) and cultured at 37°C with 5% CO_2_ for 24 h. The antibody and pseudovirus were treated before the experiment: mAbs were diluted with DMEM containing 50 μg/ml of streptomycin (strepDMEM), of which 165 μl was added to a 96-well plate in triplicate. The pseudovirus was diluted to 10000 TCID_50_/ml, and 75 μl of virus was added to each well that contained antibody and then cultured at 37°C for 1 h. Virus control contained 150 μl of strepDMEM and 75 μl of pseudovirus. Cell control contained 225 μl of strepDMEM. After cell incubation, the supernatant was discarded, 70 μl of virus-antibody mixture was added and incubated at 37°C for 24 h, and then replenished with 100 μl/well and incubated for 24 h. The cell supernatant was discarded and 50 μl of cell lysate was added into a shaking table at room temperature for 30 min, of which 30 μl was added to an optical white bottom plate. Fifty microliters of luciferase reagent were added to each well to measure luciferase activity by measuring RLU. Neutralization was calculated by an equation of [1- (average RLU of test antibody sample - average RLU of cell control)/(average RLU of virus control - average RLU of cell control)] × 100% (24).

### 2.6 Flow cytometry isolation of single B cells

To sort out MERS-CoV S2-specific single B cell, PBMCs stored in liquid nitrogen were thawed in a 37°C water bath and immediately cultured in RPMI 1640 medium supplemented with 10% FBS in an incubator with 5% CO_2_ at 37°C. To prepare fluorescent MERS-CoV S2 probes, the MERS-CoV S2 protein was conjugated to fluorescein isothiocyanate (FITC) and labeled with Alexa Fluor^®^ 647 or Alexa Fluor^®^ 488. For cell surface staining, 1×10^6^ PBMCs were first labeled using the LIVE/DEAD Fixed Blue DEAD cell staining kit (L34962, Thermo Fisher Scientific) to distinguish live and dead cells. The treated PBMCs were immunostained with antibodies that have been diluted to the optimum concentration and incubated at 4°C for 30 min. The fluorescently labeled antibodies used were as the followings: IgG PE (G18-145, BD Biosciences, USA), IgD PE/Dazzle™ 594 (IA6-2, BioLegend, USA), CD19 percp 5.5 (HIB19, BioLegend), CD27 PE-cy7 (M-T271, BioLegend), and CD3 BUV737 (SK7, BD Bioscience). Immediately after antibody staining, samples were loaded to MoFlo XDP flow cytometer (Beckman Coulter, USA) and single B cells (S2+AF647+ and S2+AF488+) was sorted into each well of 96-well plates containing 7 μl lysis buffer (10% IGEPAL, 100 mM DTT, 40 U/μl RNAase) and placed on dry ice to freeze quickly. The antibody heavy chain and light chain mRNA were reverse transcribed and nested PCR amplified (below). The frequency of sorted S2+ B cells was analyzed using FlowJo, version 10.8.1.

### 2.7 Expression and purification of antibodies

The VH and VL sequences of the IgG antibodies were amplified by RT-PCR and cloned into the vector plasmids AbVec2.0-hIgG1 (80795, Adgene, USA) for expressing the heavy chain, MapAbVec1.1-IGKC (80796, Adgene) for expressing the kappa light chain, and AbVec1.1-IGLC2 (99575, Adgene) for expressing the lambda light chain. Antibody expression plasmids (0.5 μg/ml) were co-transfected into 293F cells (1×10^6^ cells/ml) in SMM 293-TII expression medium (M293TII, SinoBiological) using PEI transfection reagent (23996-2, Polysciences, Pennsylvania, USA) and cultured for 6-7 days, and the cells and cell debris were removed by centrifugation at 4500 ×g and filtration (0.22 mm). The supernatant containing recombinant antibodies were purified using an ÄKTA express FPLC device using Protein A columns, washed with 20 ml of PBS, and eluted with glycine elution buffer (pH=2.0) into a collection tube containing Tris HCl (pH = 8.0). The purified antibody was dialyzed three times in PBS and stored at -40°C for later use. The purity of the antibodies were checked by running a polyacrylamide gel electrophoresis (PAGE) and visualized with Coomassie staining.

### 2.8 Determination of antibody binding epitopes

To distinguish whether antibodies recognize linear or discontinuous epitopes of S protein, we adopted the method previously described (21). Ninety-six-well plates were coated with MERS-CoV S2 (200 ng/well) in PBS at 4°C overnight. The plates were washed five times, and the coated-S2 was treated with or without denaturing buffer (50 μl/well; 200 mM DTT and 4% SDS in PBS) for 1 h at 37°C. After washing five times with PBS-T, the plates were blocked with blocking buffer at RT for 2 h. The mAbs (1 μg/ml) were added to the plate (100 μl/well) and incubated at 37°C for 1 h. The plates were washed five times and then analyzed for antibody binding to MERS-CoV S2 using a microplate reader (Thermo Fisher Scientific).

### 2.9 mAbs binding of synthetic MERS-CoV S2 stem peptides

To determine the regions of MERS-CoV S2 potentially bound by mAbs, peptides covering the S2 region were used as coating antigen in the indirect ELISA. Briefly, peptides 15-25 amino acids (aa) were synthesized, coated to 96-well ELISA plates (20 ng/μl, 100 μl/well), and incubated at 37°C for 2 h. The plate was then washed three times with PBS-T and blocked with a blocking buffer at room temperature for 3 h. The mAb (1 μg/ml) was added to the well at 100 μl/well and incubated at 37°C for 1 h. After washing five times, the plate was incubated with the HRP-conjugated goat anti-human IgG at 37°C for 1 h. The OD_450_ nm was measured using the microplate reader. MERS-CoV S2 (2 ng/μl) was used as a positive control, and BSA protein was used as a negative control.

### 2.10 Data analysis

The data were processed and figures were plotted using GraphPad Prism (version 8.0).

## 3 Results

### 3.1 Serum IgG antibodies from COVID-19 convalescents reacted with MERS-CoV S2 subunit

**Figure 1:**
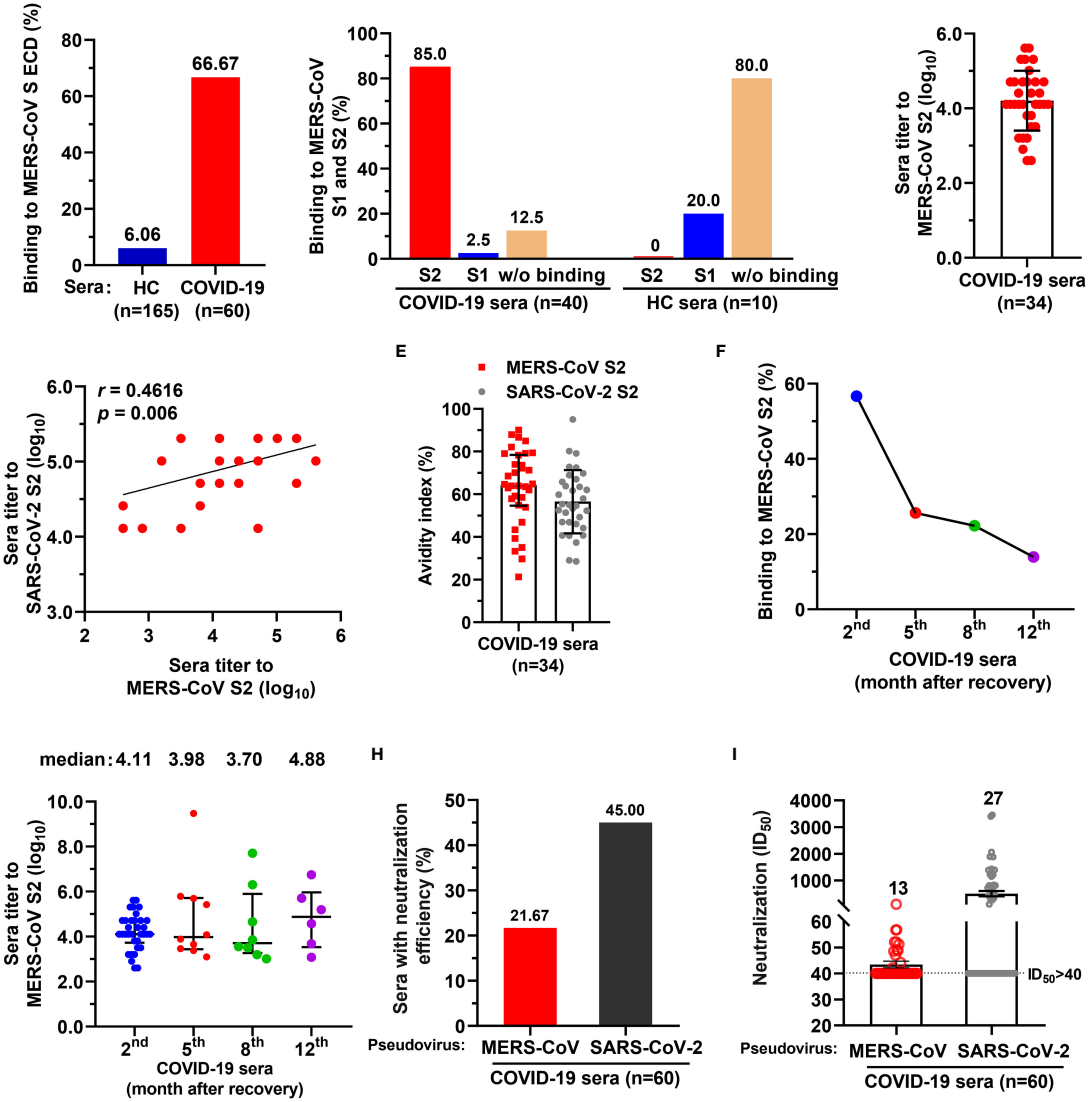
A proportion of serum antibodies from COVID-19 convalescents had binding activity to MERS-CoV spike S2 subunit and neutralized spike pseudoviruses. **(A)** A proportion of COVID-19 sera showed binding activity to MERS-CoV spike (S) extracellular domain (ECD). Sera from COVID-19 convalescents (n=60) and healthy control (HC) blood donors (n=165) (both at 1:1000 dilution) were tested for binding to MERS-CoV S ECD by ELISA. The percentage of sera binding to MERS-CoV S ECD are shown. Data are the average values from two independent experiments. **(B)** The percentage of sera from COVID-19 convalescents or HC reacted with MERS-CoV S1 and/or S2. **(C)** Endpoint titer of serum IgGs from COVID-19 convalescents against MERS-CoV S2. **(D)** Correlation coefficient of endpoint titers for antibodies reactive with MERS-CoV S2 and SARS-CoV-2 S2. Pearson’s correlation, *p*<0.05 was considered statistically significant. **(E)** Avidity of COVID-19 convalescent serum antibody to MERS-CoV S2 and SARS-CoV-2 S2. **(F)** Dynamics of COVID-19 convalescent serum antibody after discharge. Binding activity of COVID-19 serum antibody against MERS-CoV S2 subunit was examined after discharge, at the 2^nd^ month (n=60), the 5^th^ month (n=39), the 8^th^ month (n=36), and 12^th^ month (n=43). **(G)** Endpoint titers of COVID-19 convalescent serum antibody for MERS-CoV S2 after discharge from hospital. The convalescent sera were tested at the 2^nd^ month (n=34), the 5^th^ month (n=10), the 8^th^ month (n=8), and 12^th^ month (n=6). One-way ANOVA with Tukey’s *post hoc* test was used. **(H)** The percentage of COVID-19 convalescent serum IgG antibody with neutralizing activity for MERS-CoV and SARS-CoV-2 spike pseudoviruses. ID_50_>40 was considered positive for neutralization (21.67% [13/60] and 45% [27/60], respectively). **(I)** Neutralization titers of 13 COVID-19 convalescent serum antibodies for MERS-CoV and SARS-CoV-2 spike pseudoviruses. ID_50_>40 was considered positive for neutralization. For panels *C*, *D*, and *G*, endpoint titers were log_10_-transformed; for panels *C*, *E*, *G* and *I*, data are median ± IQR (25–75%) and the error bars indicate median with interquartile range (IQR).

To examine whether SARS-CoV-2 infection elicit antibodies reactive to MERS-CoV, we assessed COVID-19 convalescent sera (n=60, the 2^nd^ month after discharge) using IgG-specific ELISAs against MERS-CoV spike (S) protein extracellular domain (ECD). Healthy donor control blood (HC, n=165) were collected before the COVID-19 pandemic (**Supplementary Table S1**). We found that 6.06% (10/165) of healthy sera showed binding activity to the MERS-CoV S ECD, while 66.67% (40/60) of COVID-19 convalescent sera had binding activity (**Figure 1A**). The binding activity of HC sera may result from previous exposures to seasonal coronavirus, eliciting cross immunity with other coronaviruses. Following SARS-CoV-2 infection, cross-reactive memory B cells were activated and secreted antibodies responsive to MERS CoV S ECD (25). Further, 20% (2/10) of the S ECD-binding healthy sera reacted with S1 subunit, but none bound with S2 (**Figure 1B**). In contrast, 85% (34/40) of S ECD-binding convalescent sera had binding activity to S2, and only 2.5% (1/40) had binding with S1 (**Figure 1B**). We also determined the endpoint titers of 34 IgG antibodies targeting MERS-CoV S2 (median, 1:12800 dilution; [log_10_ = 4.11; interquartile range [IQR], 3.81–4.71]) (**Figure 1C**) and found that they positively correlated with the titers of SARS-CoV-2 S2-targeting antibodies (r=0.4616, p=0.006) (**Figure 1D**). All 34 IgG antibodies had avidity values similarly for MERS-CoV S2 (median, 64.30; IQR, 55.59-77.18) and SARS-CoV-2 S2 SARS-CoV-2 (median, 55.04; IQR, 46.91-65.76) (**Figure 1E**).

To evaluate the dynamics of COVID-19 serum IgGs, we examined the binding activity and endpoint titers of the sera against MERS-CoV S2 at the 5^th^ month (n=36), 8^th^ month (n=39), and 12^th^ month (n=43) after discharge. The number of MERS-CoV S2 reactive sera decreased sharply from the 2^nd^ month to the 5^th^ month, and only 25.64% (10/39) of sera remained reactive with S2 at the 5^th^ month (**Figure 1F**). The number of S2-binding sera continually decreased to 13.95% (6/43) from the 5^th^ to the 12^th^ month (**Figure 1F**). The endpoint titers of reactive sera were 4.11, 3.98, 3.70, and 4.88 for the 2^nd^, 5^th^, 8^th^, and 12^th^ months, respectively (log_10_ transformed; **Figure 1G**).

Next, we evaluated the neutralization of COVID-19 convalescent sera against MERS-CoV spike pseudovirus and found that 21.67% (13/60) of sera neutralized MERS-CoV pseudovirus (defined by 50% inhibitory dilution [ID_50_] of 1.71, namely 1:51.26 dilution without log_10_ transformed; IQR, 1.69-1.75, namely 1:48.55-1:56.71 dilution) (**Figure 1H** and **Supplementary Table S2**), with ID_50_ ranging from 42 to 108 (**Figure 1I**). In contrast, 45% (27/60) of sera showed neutralization with SARS-CoV-2 (Figure 1H), with ID50 ranging from 113 to 3457 (**Figure 1I**). Taken together, these data suggest that a small proportion of the general population elicited antibodies with binding activity to MERS-CoV S ECD, of which 80% did not bind to S1 and S2 fragments; 85% of COVID-19 convalescent serum antibodies reacted with MERS-CoV S2. Few of COVID-19 convalescent IgG antibody could neutralize MERS-CoV spike pseudovirus.

### 3.2 mAbs constructed from COVID-19 convalescent memory B cells showed binding activity with MERS-CoV spike

**Figure 2:**
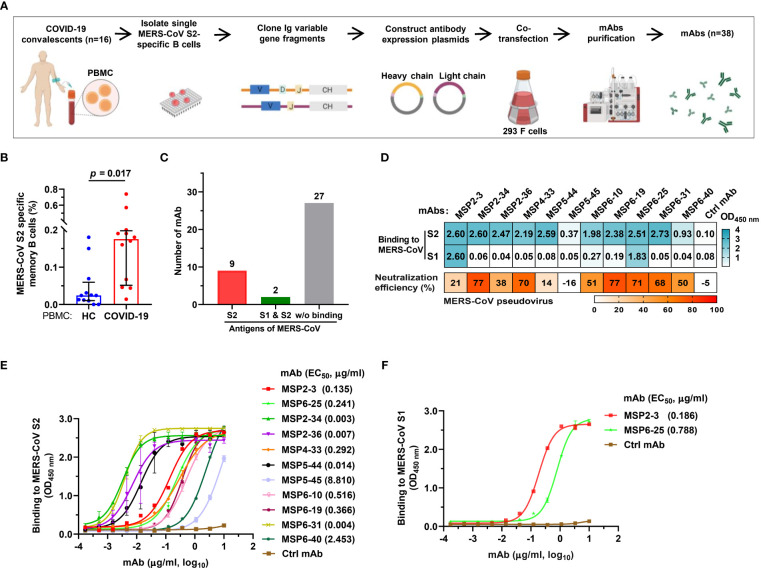
Isolation of MERS-CoV S2-specific antibodies from COVID-19 convalescent memory B cells. **(A)** The workflow of producing human monoclonal antibodies (mAbs) by cloning antibody genes from memory B cells. The memory B cells were isolated from PBMCs of 16 COVID-19 convalescents, and the variable regions of the heavy and light chains of antibodies (VH, VL/VK) were amplified from single B cells by RT-PCR and cloned into antibody expression vector plasmids. The selected VH and VL/VK clones were sequenced and co-transfected into 293F cells. The supernatants were taken after 6-7 days of culture, and the monoclonal antibodies were purified by ÄKTA Purifier. **(B)** The frequency of MERS-CoV spike S2-specific memory B cells in healthy blood donors and COVID-19 convalescents. Twelve experiments of cell screening were performed, and one healthy sample (negative control) was included in each experiment. Unpaired *t*-test was used to compare the difference between two groups, *p*<0.05 was considered for statistical significance (GraphPad Prism, version 8.0). **(C)** mAbs binding to MERS-CoV S1 and/or S2. A total of 38 mAbs constructed in this study were tested. **(D)** The binding of mAbs (1 μg/ml) to MERS-CoV S1 or S2 (cyan). The OD_450nm_>0.3 was defined as positive. Neutralization of mAbs (50 μg/ml) against MERS-COV pseudovirus was defined as >50% infection reduction. **(E, F)** ELISA curves of antibodies binding to MERS-CoV S1 and S2. An unrelated anti-HCV mAb, 2HCV5, was used as negative control (Ctrl mAb). Error bars indicate the mean ± standard error of mean (SEM), and the data represent technical replicates of at least two independent experiments. Data were analyzed and plotted by GraphPad Prism (version 8.0).

The binding and neutralizing activity of COVID-19 convalescent sera with MERS-CoV urged us to construct mAbs with dual-reactivity from the convalescent memory B cells. We isolated PBMCs from 16 COVID-19 convalescents using MERS-CoV S2 probes and sorted single memory B cells (CD19+/CD3-/CD27+/IgD-/IgG+) (**Figure 2A** and **Supplementary Table S3**). Flow cytometry analysis revealed that 0.18% of COVID-19 PBMCs were memory B cells specific to MERS-CoV S2, while only 0.02% was found in healthy control PBMCs (p = 0.017) (**Figure 2B** and **Figure S1**). The variable heavy (VH) and variable light (VL) chains of antibodies were amplified by RT-PCR, cloned, and expressed in 293F cells. A total of 38 mAbs were purified and tested for reactivity with MERS-CoV S2 proteins by single concentration qualitative ELISA (**Figure 2A** and **Figure S2**). The results showed that 11 mAbs showed binding activity to MERS-CoV S2, of which 2 mAbs also bound to S1 (**Figures 2C, D**).

To further determine the binding efficiency, the 11 mAbs were made into 3-fold serial dilutions and quantitatively detected by a MERS-CoV S2-coated ELISA. The results showed that 4 mAbs had potent binding activity (MSP2-34, MSP2-36, MSP5-44, and MSP6-31; EC_50_, 0.003–0.014 μg/ml), 5 mAbs showed strong binding (MSP2-3, MSP4-33, MSP6-10, MSP6-19, and MSP6-25; EC_50_, 0.135 - 0.516 μg/ml), and 1 mAb had moderate binding (MSP6-40; EC_50_, 2.453 μg/ml), and 1 mAb showed weak binding activity (MSP5-45; EC_50_, 8.810 μg/ml) to MERS-CoV S2 (**Figure 2E**). In addition, we also tested the binding activity to MERS-CoV S1 and found that 2 of the 11 mAbs were capable of binding with S1 (MSP2-3, EC_50_, 0.186 μg/ml; MSP6-25, EC_50_, 0.788 μg/ml) (**Figure 2F**).

Next, we tested neutralization effect on MERS-CoV pseudovirus and found that seven mAbs, MSP2-34, MSP4-33, MSP6-10, MSP6-19, MSP6-25, MSP6-31 and MSP6-40 could neutralize MERS-CoV spike pseudovirus, with infection reduction by ~50%-77% when mAbs were used up to 50 μg/mL (**Figure 2D**). Thus, 11 mAbs had weak neutralization effect against MERS-CoV pseudovirus.

To further characterize these antibodies, we analyzed the coding sequences of 11 mAbs using IgBLAST tool (http://www.ncbi.nlm.nih.gov/igblast/) (**Table 1**). The VH sequences of mAbs were from four gene classes, VH1, VH2, VH3, and VH4, and most antibodies were composed of VH3 heavy chain subgroup and kappa (κ) light chains. The average length of the antibody complementarity-determining region 3 of the heavy chain (H-CDR3) and light chains (L-CDR3) were 15 and 10 amino acids, respectively. The frequency of somatic mutations in VH and VL nucleotide sequences was within the normal range, from 5% to 14% for VH and from 5% to 13% for VL, with exception of MSP6-25 and MSP6-40, whose mutation frequency of VH and VL was from 2% to 5% (**Table 1**). Collectively, we have constructed a panel of mAbs from COVID-19 convalescent single B cell, of which most had reactivity with MERS-CoV S2 subunit.

**Table 1:** Genetic characteristics of mAbs with reactivity against MERS-COV S1 and/or S2.

mAbs	MERS-CoV, EC_50_(μg/mL)		Binding domain	Heavy chain			Light chain		
	S1	S2		VH gene	H-CDR3 (bp)	VH identify (%)	VL gene	L-CDR3 (bp)	VL identify (%)
MSP2-3	0.186	0.135	S1 and S2	IGHV3-74	8	89.2	IGLV2-14	10	92.9
MSP2-34	–	0.003	S2	IGHV1-46	13	91.1	IGKV1-27	11	94.1
MSP2-36	–	0.007	S2	IGHV4-59	19	86.4	IGLV2-8	10	96.9
MSP4-33	–	0.292	S2	IGHV2-5	15	90	IGKV4-1	9	86.7
MSP5-44	–	0.014	S2	IGHV3-11	20	92.5	IGKV3-11	11	90.9
MSP5-45	–	8.810	S2	IGHV3-7	16	91.6	IGKV2-30	9	95.7
MSP6-10	–	0.516	S2	IGHV3-7	16	89.2	IGKV3-20	9	89.8
MSP6-19	–	0.366	S2	IGHV3-48	12	88.2	IGLV1-44	11	92.8
MSP6-25	0.788	0.241	S1 and S2	IGHV3-11	19	97.6	IGLV2-23	13	96.2
MSP6-31	–	0.004	S2	IGHV3-30	12	94.6	IGKV1-9	9	95.1
MSP6-40	–	2.453	S2	IGHV3-7	16	95.6	IGKV2-30	9	95.3

ELISA-based half-maximal effective concentrations (EC_50_) and genetic characterization of mAbs. Heavy and light chain genes, % difference relative to germline sequence and CDR3 length were analyzed using the IgBlast website (http://www.ncbi.nlm.nih.gov/igblast/).

### 3.3 Cross-reactivity of mAbs with all seven human coronaviruses

**Figure 3:**
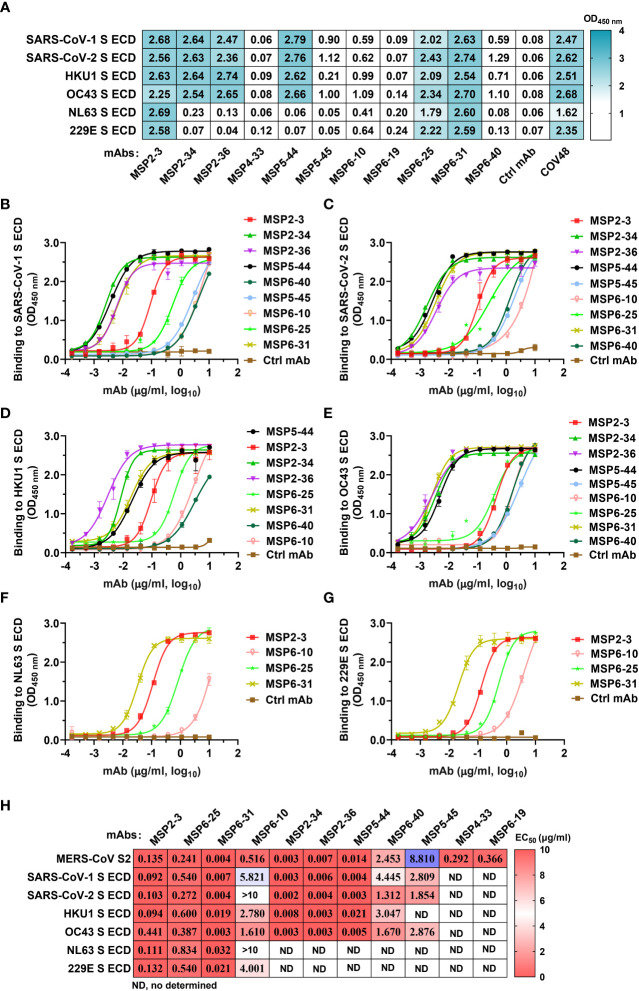
Cross-reactivity of mAbs isolated from COVID-19 convalescents with other hCoVs. **(A)** Cross-reactivity of mAbs with seven human coronaviruses (hCoVs) at a concentration of 1 μg/ml. The positive control COVID-19 serum COV48 (1:1000 dilution) and unrelated anti-HCV mAb 2HCV5 (Ctrl mAb) were used as positive and negative controls, respectively. OD_450 nm_>0.3 was defined as a positive reaction. **(B–G)** ELISA binding curves of mAbs reactive to hCoVs S ECD (panel A). The S ECDs of SARS-CoV-1, SARS-CoV-2, HKU1, OC43, NL3, and 229E were coated at 2 μg/ml. An unrelated anti-HCV mAb 2HCV5 (Ctrl mAb) was used as a negative control. Data represent the mean ± SEM of two replicates. **(H)** The EC_50_ of mAbs binding to various spike ECD proteins. Data are calculated from panes **(B–G)**. ND, no or low reaction in single dose screening **(A)**, thus it was not determined by dose-dependent ELISA. Data were analyzed and plotted by GraphPad Prism (version 8.0).

To assess whether these mAbs react with other human coronaviruses, we screened the binding of the 11 mAbs (1 μg/ml) with spike trimers of six coronaviruses by ELISA. The results showed that MSP2-3, MSP6-25, and MSP6-31 efficiently bound to all six spike proteins (OD_450_ nm >1.7), MSP2-34, MSP2-36, and MSP5-44 bound to each of SARS-CoV-1, SARS-CoV-2, OC43, and HKU1, while other mAbs showed weak or no binding to these viruses (**Figure 3A**). Moreover, dose-dependent ELISA revealed that MSP2-3, MSP6-25, and MSP6-31 showed broad and potent cross-reactivity with all 6 spike ECDs and MERS-CoV S2 (EC_50_, 0.003-0.540 μg/ml) (**Figures 3B–H**). MSP6-10 bound potently MERS-CoV S2 and weakly with 4 spike ECDs (EC_50_ >0.55 μg/ml), and had no binding with SARS-COV-2 and NL63 (EC_50_>10 μg/ml) (**Figures 3B–G**). MSP2-34, MSP2-36, and MSP5-44 displayed cross-reactivity with the spike ECDs of SARS-CoV-1, SARS-CoV-2, OC43, and HKU1 (**Figures 3B–E, H**). MSP4-33 and MSP6-19 did not react with other coronaviruses but MERS-CoV S2 (**Figure 3A** and 3H). MSP6-40 and MSP5-45 had weak cross-reaction with MERS-CoV S2, SARS-CoV-1, SARS-CoV-2, and OC43 (**Figures 3A–E, H**).

### 3.4 Binding epitope analysis of mAbs for MERS-CoV S2

**Figure 4:**
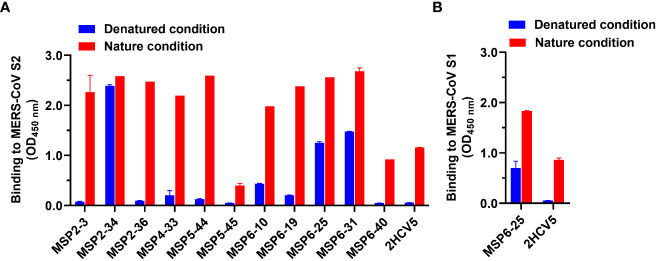
Analysis of binding epitopes of mAbs. **(A)** Binding of mAbs (1 μg/ml) to MERS-CoV S2 in non-treated nature condition and heat-denatured condition (37°C, 1 h, in the presence of SDS and DTT, blue). An unrelated conformational anti-HCV mAb 2HCV5 was used as a control, to which the coating antigen was HCV E2 protein. **(B)** Binding of antibody MSP6-25 (1 μg/ml) to MERS-CoV S1 in nature condition and in heat-denatured condition in the presence of SDS and DTT (blue). Data were analyzed and plotted by GraphPad Prism (version 8.0).

Further, we proceeded to determine the antibody binding epitopes. We analyzed the binding activity of 11 mAbs against heat-denatured and untreated natural MERS-CoV S2 antigens in the presence of SDS and DTT by ELISA. The results showed that MSP2-34, MSP6-25, and MSP6-31 had good binding to both denatured and natural antigens, while other antibodies reacted with the non-denatured S2 only. These results suggest that MSP2-34, MSP6-25, and MSP6-31 recognized a continuous linear epitope in MERS-CoV S2, while the other mAbs may bind to conformational epitopes (**Figure 4A**). Since MSP6-25 showed binding to both MERS-CoV S1 and S2 (**Figures 2D–F**), we analyzed the binding epitope of MSP6-25 in the S1 and found that MSP6-25 recognizes a linear sequence of MERS-CoV S1 (**Figure 4B**).

**Figure 5:**
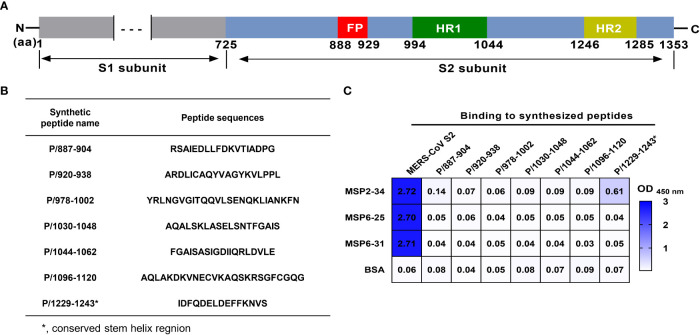
Binding of mAbs to different synthetic MERS-CoV S2 peptides. **(A)** Diagram of MERS-CoV S protein. Amino acid (aa) positions are referred to the sequence of strain HCoV-EMC (GenBank: AFS88936.1). **(B)** Amino acid sequence of peptides. **(C)** Binding of mAbs MSP2-34, MSP6-25, and MSP6-31 (1 μg/ml) to synthetic peptides (20 ng/μl) by ELISA. MERS-CoV S2 subunit (2 μg/ml) was positive control. BSA was used as negative control.

Next, we attempted to analyze which regions of MERS-CoV S2 were recognized by mAbs MSP2-34, MSP6-25, and MSP6-31. We synthesized seven peptides covering the conserved regions in the S2 subunits of seven hCoVs (**Figures 5A, B**) and performed ELISA with three mAbs (**Figure 5C** and **Figure S3**). The results showed that only MSP2-34 had weak binding to peptide P/1229-1243 (OD_450_ = 0.61) (**Figure 5C**), located in the stem helix region of MERS-CoV S2 upstream of heptapeptide repeat 2 (HR2) region (**Figure 5A**). MSP6-25 and MSP6-31 had no binding to peptides (**Figure 5C**).

## 4 Discussion

In recent years, sporadic cases of MERS-CoV were recorded in the Middle East, increasing the risk of spreading to other countries and regions. To date, there are not any MERS-CoV vaccine or antiviral drugs available for the prevention and treatment of MERS (26). SARS-CoV-2 is rampant around the world, with rapid emergence of various variants, causing tremendous losses to global socio-economies and public health. Therefore, there is an urgent need to develop approaches for therapy, control, and prevention of MERS-CoV and SARS-CoV-2, as well as other coronaviruses. In this study, we identified 11 out of 38 mAbs from COVID-19 convalescent individuals had binding activity to MERS-CoV S2, and 5 mAbs showed neutralizing activity for MERS-CoV spike pseudovirus. Moreover, 9 of the 11 mAbs also showed binding with SARS-CoV-2 spike ECD, as well as the other three or five hCoVs. The mAbs with broad-spectrum binding activity for CoVs contribute to the diagnosis and research for MERS-CoV and SARS-CoV-2, as well as other coronaviruses.

Coronaviruses enter the host cells through the binding of spike protein to cellular receptors. Therefore, targeting the spike protein represents the main mechanism for antibody neutralization of coronaviruses. The coronavirus S protein consist of S1 and S2 subunits; S1 subunit includes the receptor binding domain important for receptor recognition (DPP4 in the case of MERS-CoV, also known as CD26), while S2 subunit contains the fusion peptide, heptad repeats (HR) 1 and 2, and a transmembrane structural domain. These S2 domains are required for membrane fusion of the virus and host cells (27). Theoretically, antibodies targeting the conserved epitopes of coronavirus S protein have a high chance to exhibit broad neutralizing activity against multiple coronaviruses, and such antibodies are attractive for treatment and for pan-coronavirus vaccine design (28). Here, we first analyzed the serological responses of COVID-19 convalescent individuals and found that SARS-CoV-2 infection enhanced the cross-reactivity of serum antibodies to MERS-CoV spike, mainly targeting S2 subunit. This notion is supported by the positive correlation of the serum antibody titers to MERS-CoV S2 and SARS-COV-2 S2 (**Figure 1D**). However, it should be noted that such correlation may be partially due to the similarities in structure and sequence between SARS-CoV-2 S2 and MERS-CoV S2 that share a 45% sequence homology in the conserved S2 region. Of serum antibodies, only 21.67% (13/60) showed neutralization effect against MERS-CoV spike pseudovirus, suggesting that antibodies binding to S2 may not necessarily interfere with the binding of the DPP4 receptor (**Figure 1H**). Together, these data showed that COVID-19 convalescent sera could serve as better candidates for construction of mAbs neutralizing or binding to other coronaviruses.

We constructed 38 mAbs from memory B cells and found 11 mAbs with binding activity to MERS-CoV S2. Three of them, MSP2-3, MSP6-25, and MSP6-31 showed broad and potent binding activity to all other hCoVs, while MSP2-34, MSP2-36, and MSP5-44 bound only to beta-coronaviruses (**Figure 3H**). In addition, 5 mAbs (MSP2-34, MSP4-33, MSP6-19, MSP6-25, and MSP6-31) showed weak neutralization effect to MERS-CoV pseudovirus. Given the reactivity to multiple coronaviruses, these mAbs, singly or in combination, could be useful in the development of diagnosis methods distinguishing MERS-CoV and other coronaviruses.

Antibody binding epitopes could be linear and conformational, with about 10% of antibodies recognizing linear epitopes (29). Three mAbs recognized linear epitopes, and 8 mAbs bound to conformational epitopes (**Figure 4A**). It has been reported that antibodies targeting the S2 stem region could neutralize MERS-CoV pseudovirus, as the antibody binding obstructed the formation of the hexa-helix bundle of spike proteins during fusion step of virus entry (21, 22). The other antibody 76E1 was reported to target the conserved S2’ site of SARS-CoV-2 spike protein and inhibits S2’ cleavage, thus blocking membrane fusion and virus entry (30). However, antibodies MSP2-34, MSP6-25, and MSP6-31, targeting linear epitopes, bound efficiently to multiple coronaviruses (**Figures 2**–**4**), though they had a low neutralization efficiency to MERS-CoV spike pseudovirus (**Figure 2D**). Several key residues have been reported to be involved in antibody-antigen interactions; they are imbedded fully or partially in pre-fusion S trimers and go through conformational changes from pre-fusion to post-fusion states of the viral S-trimers, thus leading to epitope exposure and binding of neutralizing antibodies (30–32). The weaker neutralization of MSP2-34, MSP6-25, and MSP6-31 may result from a limited epitope exposure of the S protein. However, further analysis of these S2-specific antibodies is needed.

Although mechanism of action of non-neutralizing antibodies is complex or unknown, nine broadly reactive antibodies constructed here are of great value in the diagnosis and prevention of early viral infections. Binding of antibody Fab fragments to viral antigens forms antigen-antibody immune complexes, and the antibody Fc fragment mediates antiviral function by binding to the Fc receptor (FcγRs) on natural immune cells through antibody-dependent cellular cytotoxicity (ADCC), complement-dependent cytotoxicity (CDC), or antibody-dependent cell phagocytosis (ADCP) (33, 34). It has been shown that non-neutralizing antibodies that target Ebola virus and respiratory syncytial virus could mediate ADCC effects and lead to a promising outcome in virus-infected mice (35, 36). In addition, non-neutralizing antibodies binding to conserved viral epitopes could trigger ADCC and thus result in a broad cross-protective response (37, 38). Due to the antiviral pressure, viruses tend to evade neutralizing antibody responses. Thus, the cross-protective immune responses that non-neutralizing antibodies may have would be particularly important.

In summary, we generated mAbs with specific binding activity to all seven human coronaviruses. These antibodies will facilitate future studies on neutralizing and binding antibodies against multiple coronaviruses, as well as vaccine design for pan-coronaviruses. Given broad-reactivity of these antibodies, more mAbs from COVID-19 convalescents, epitope analysis, and structure-guided antibody engineering will be of great interest.

## Data availability statement

The original contributions presented in the study are included in the article/**Supplementary Material**. Further inquiries can be directed to the corresponding authors.

## Author contributions

YP, YL, YH, XQ, and Y-PL contributed to the study design and data interpretation. YP, YL, YH, FC, QW, JY, JC, ST JZ, RH, XQ, and Y-PL contributed to clinical management, patient recruitment and data collection. YP,YL, YH, FC, JZ, RH, YW, MB, TL, and WL contributed to statistical analysis and data visualization. YP, YL, Y-PL drafted the manuscript. MB, XQ and Y-PL contributed to revision of the manuscript for important intellectual content. All authors contributed to the article and approved the submitted version.

## Supplementary material

Supplementary Figure 1 Gating strategy for isolating MERS-CoV spike S2-specific memory B cells from COVID-19 convalescents. PBMC of COVID-19 convalescents were first stained with LIVE/DEAD and CD3/CD19 -specific antibodies to exclude T cells and monocytes. Then, CD27/IgD/IgG antibodies were used to select mature B cells. Two probes MRES-CoV S2-AF488 and S2-AF647 were used to select MERS-CoV S2-specific memory B cells. Single B cell position for S2^+^/AF647^+^ and S2^+^/AF488^+^ was sorted into 96-well plates for construction of mAbs.

Supplementary Figure 2 The purity of mAbs by using PAGE. The mAbs constructed in this study were purified by AKTA and PFLC approaches. The purity of mAbs was checked by PAGE with Coomassie staining. The evelen mAbs with binding to MERS-CoV S2 are showen.

Supplementary Figure 3 Amino acid sequence alignment of the stem region of spike proteins of seven hCoVs. The alignment was performed using spike protein sequences: MERS-CoV (GenBank accession number: AFS88936.1), SARS-CoV-2 (NCBI Reference Sequence: YP_009724390.1), SARS-CoV (NCBI Reference Sequence: NP_828851.1), OC43 (GenBank: AVR40344.1), HKU1 (UniProtKB/Swiss-Prot: Q0ZME7.1), NL63 (GenBank: APF29071.1), and 229E (GenBank: APT69883.1). Sequence alignment was performed using CLUSTALW (Clustal Omega < Multiple Sequence Alignment < EMBL-EBI) and visualized using ESPript 3.0 (ESPript 3.x/ENDscript 2.x).
