# Supplementary figures and images for: Monoclonal antibodies constructed from COVID-19 convalescent memory B cells exhibit potent binding activity to MERS-CoV spike S2 subunit and other human coronaviruses
Source: Front Immunol. 2022 Dec 22;13:1056272. doi: 10.3389/fimmu.2022.1056272 (PMC9813381; doi:10.3389/fimmu.2022.1056272)

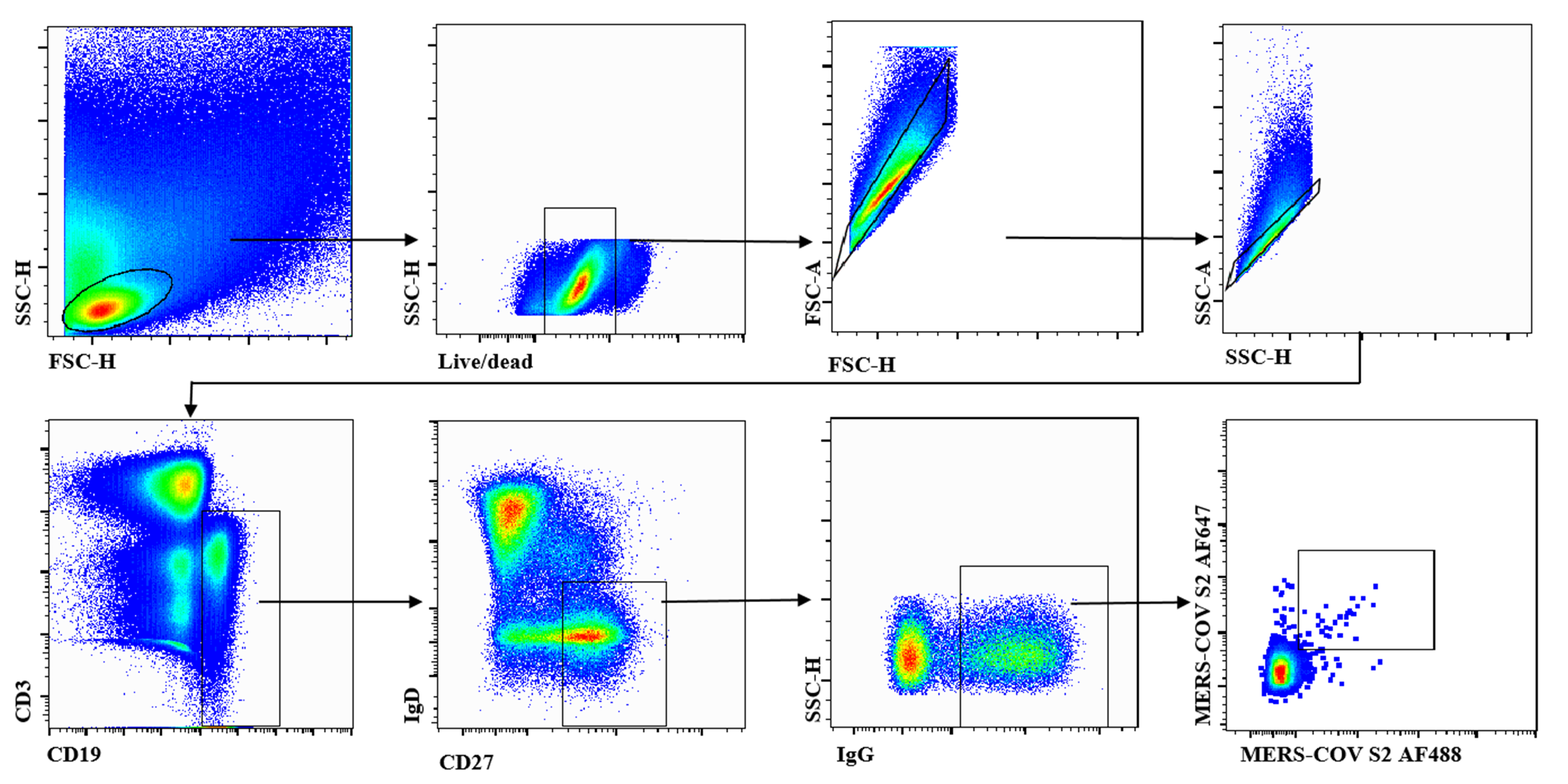

Supplement: Supplementary Figure 1 — Gating strategy for isolating MERS-CoV spike S2-specific memory B cells from COVID-19 convalescents. PBMC of COVID-19 convalescents were first stained with LIVE/DEAD and CD3/CD19 -specific antibodies to exclude T cells and monocytes. Then, CD27/IgD/IgG antibodies were used to select mature B cells. Two probes MRES-CoV S2-AF488 and S2-AF647 were used to select MERS-CoV S2-specific memory B cells. Single B cell position for S2+/AF647+ and S2+/AF488+ was sorted into 96-well plates for construction of mAbs. [file Image_1.tif]

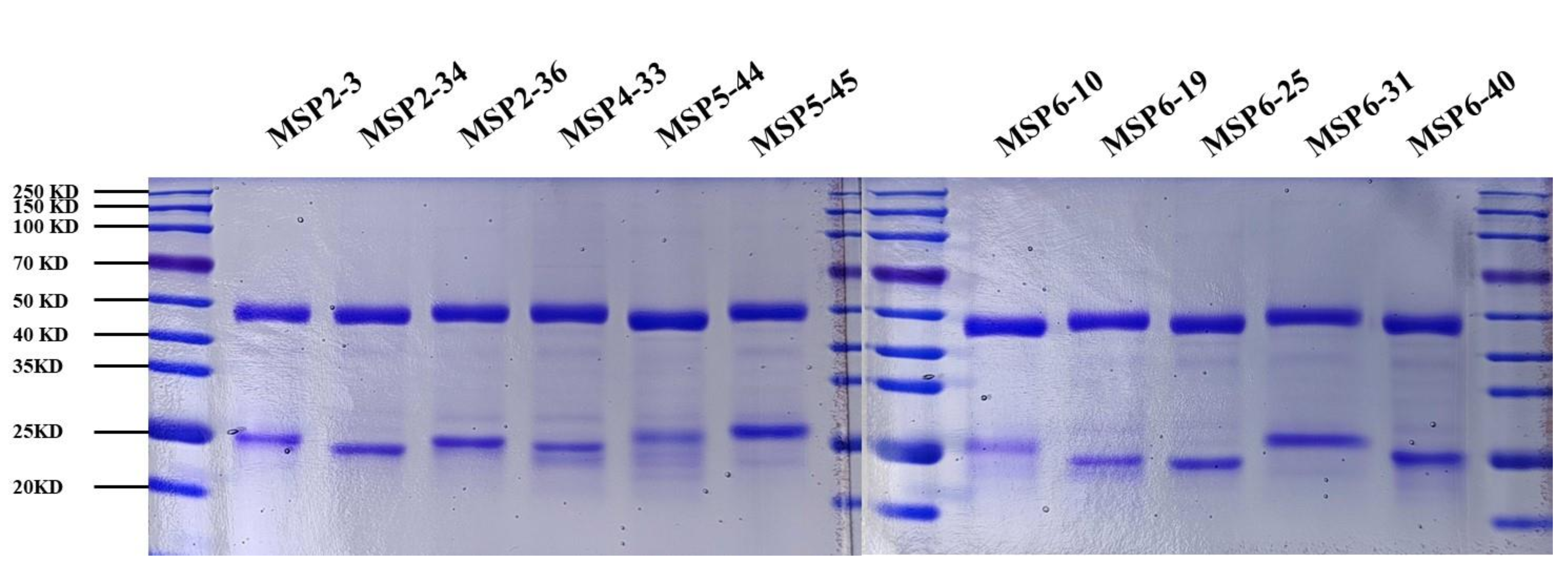

Supplement: Supplementary Figure 2 — The purity of mAbs by using PAGE. The mAbs constructed in this study were purified by AKTA and PFLC approaches. The purity of mAbs was checked by PAGE with Coomassie staining. The evelen mAbs with binding to MERS-CoV S2 are showen. [file Image_2.tif]
