# Supplementary material for: Monoclonal antibodies constructed from COVID-19 convalescent memory B cells exhibit potent binding activity to MERS-CoV spike S2 subunit and other human coronaviruses
Source: Front Immunol. 2022 Dec 22;13:1056272. doi: 10.3389/fimmu.2022.1056272 (PMC9813381; doi:10.3389/fimmu.2022.1056272)
Supplement: Supplementary file 4 [file Table_1.doc]

**Supplementary Table S1. Basic information of healthy donor (general population) and COVID-19 convalescents**

| **Cohort** | **Number** | **Severe/non-severe** | **Sex (F/M)** | **Age (range)** | **Day hospitalized (range)** | **Endpoint titers to MERS-CoV** | | **Avidity to MERS-CoV** | | **Neutralization titers to high pathogenic CoVs** | | | **Cross reactivity** | | | |
| --- | --- | --- | --- | --- | --- | --- | --- | --- | --- | --- | --- | --- | --- | --- | --- | --- |
|  |  |  |  |  |  | **S1** | **S2** | **S1** | **S2** | **MERS-CoV** | **SARS-CoV-2** | **SARS-CoV** | **MERS-CoV S1** | **MERS-CoV S2** | **SARS-CoV-2 S** | **SARS-CoV S** |
| **COVID-19 convalescents** | **60** | **16/44** | **29/31** | **43(30-51.5)** | **21(17-25)** | **400(400-400)** | **1600(400-16000)** | **ND** | **64.30(55.59-77.18)** | **40(40-40)** | **40(40-725.20)** | **40(40-121.16)** | **1.67%** | **56.67%** | **96.67%** | **96.67%** |
| **COVID-19 sera neutralizing MERS-CoV peudovirus** | **13** | **6/7** | **4/9** | **43(38.5-53.5)** | **26.5(21-29.5)** | **400(400-400)** | **12800(3200-51200)** | **ND** | **63.92(55.11-79.02)** | **51.26(48.55-56.71)** | **1306.77(726.63-1940.04)** | **164.48(40-416.65)** | **0.00%** | **100.00%** | **100.00%** | **100.00%** |
| **General population** | **165** | **-** | **75/90** | **39(31-51)** | **-** | **ND** | **ND** | **ND** | **ND** | **ND** | **ND** | **ND** | **1.21%** | **0.00%** | **6.67%** | **6.06%** |

**“ND” indicates not detected.**
