# Supplementary material for: Monoclonal antibodies constructed from COVID-19 convalescent memory B cells exhibit potent binding activity to MERS-CoV spike S2 subunit and other human coronaviruses
Source: Front Immunol. 2022 Dec 22;13:1056272. doi: 10.3389/fimmu.2022.1056272 (PMC9813381; doi:10.3389/fimmu.2022.1056272)
Supplement: Supplementary file 5 [file Table_2.doc]

**Supporting Table S2: Basic information for COVID-19 convalescents, whose sera neutralized MERS-CoV spike pseudovirus**

| **Patient ID** | **Severe/non-severe** | **Sex (F/M)** | **Age (years)** | **Day hospitalized** | **Endpoint titer to MERS-CoV** | | **Avidity to MERS-CoV** | | **Neutralization titer**  **to high pathogenic CoVs#** | | | **Cross reaction** | | | |
| --- | --- | --- | --- | --- | --- | --- | --- | --- | --- | --- | --- | --- | --- | --- | --- |
|  |  |  |  |  | **S1*** | **S2** | **S1** | **S2** | **SARS-CoV-2** | **SARS-CoV** | **MERS-CoV** | **SARS-CoV-2 S1** | **SARS-CoV-2 S2** | **SARS-CoV S1** | **SARS-CoV S2** |
| **CoV-8** | **Severe** | **Male** | **47** | **21** | **<400** | **409600** | **NA** | **86.67** | **728** | **253** | **108** | **+** | **+** | **-** | **+** |
| **CoV-16** | **Severe** | **Male** | **43** | **21** | **<400** | **25600** | **NA** | **78.26** | **2067** | **40** | **42** | **+** | **+** | **-** | **+** |
| **CoV-20** | **Non-severe** | **Female** | **39** | **19** | **<400** | **12800** | **NA** | **46.89** | **722** | **40** | **49** | **+** | **+** | **+** | **+** |
| **CoV-38** | **Non-severe** | **Female** | **28** | **28** | **<400** | **12800** | **NA** | **39.29** | **269** | **40** | **49** | **+** | **+** | **+** | **+** |
| **CoV-47** | **Non-severe** | **Male** | **42** | **25** | **<400** | **3200** | **NA** | **90.04** | **576** | **376** | **47** | **+** | **+** | **+** | **+** |
| **CoV-48** | **Severe** | **Male** | **53** | **29** | **<400** | **204800** | **NA** | **87.95** | **3457** | **513** | **57** | **+** | **+** | **+** | **+** |
| **CoV-50** | **Non-severe** | **Male** | **36** | **18** | **<400** | **25600** | **NA** | **33.28** | **813** | **40** | **52** | **+** | **+** | **-** | **+** |
| **CoV-56** | **Non-severe** | **Male** | **52** | **22** | **<400** | **409600** | **NA** | **79.02** | **1433** | **420** | **51** | **+** | **+** | **-** | **+** |
| **CoV-68** | **Severe** | **Male** | **81** | **30** | **<400** | **51200** | **NA** | **59.11** | **1225** | **76** | **44** | **+** | **+** | **+** | **+** |
| **CoV-69** | **Severe** | **Female** | **84** | **35** | **<400** | **1600** | **NA** | **57.85** | **1898** | **40** | **52** | **+** | **+** | **-** | **+** |
| **CoV-71** | **Severe** | **Male** | **54** | **28** | **<400** | **1600** | **NA** | **63.53** | **3397** | **476** | **57** | **+** | **+** | **+** | **+** |
| **CoV-74** | **Non-severe** | **Female** | **NA** | **NA** | **<400** | **3200** | **NA** | **63.92** | **NA** | **NA** | **49** | **+** | **+** | **+** | **+** |
| **CoV-75** | **Non-severe** | **Male** | **38** | **32** | **<400** | **12800** | **NA** | **70.14** | **1389** | **416** | **70** | **+** | **+** | **-** | **+** |

*, the endpoint titer below 400 (namely 1:400 dilution) was considered non-binding activity.

#, only the samples with ID_50_>40 to MERS-CoV are shown.

CoV, COVID-19 convalescents. NA, not analyzed.
