# Supplementary material for: Monoclonal antibodies constructed from COVID-19 convalescent memory B cells exhibit potent binding activity to MERS-CoV spike S2 subunit and other human coronaviruses
Source: Front Immunol. 2022 Dec 22;13:1056272. doi: 10.3389/fimmu.2022.1056272 (PMC9813381; doi:10.3389/fimmu.2022.1056272)
Supplement: Supplementary file 6 [file Table_3.doc]

**Supporting table3: Basic information of COVID-19 convalescents used in flow cytometry for memory B cells**

| **Patient ID** | **Severity of disease** | **Sex** | **Age (years)** | **Day hospitalized** | **Endpoint titer** | **Avidity** | **Neutralization titer** |
| --- | --- | --- | --- | --- | --- | --- | --- |
|  |  |  |  |  | **MERS-CoV S2** | **MERS-CoV S2** | **MERS-CoV S2** |
| **CoV-8** | **Severe** | **Male** | **47** | **21** | **409600** | **86.67** | **107.65** |
| **CoV-9** | **Non-severe** | **Male** | **44** | **21** | **1600** | **64.60** | **<40** |
| **CoV-16** | **Severe** | **Male** | **43** | **21** | **25600** | **78.26** | **42.23** |
| **CoV-19** | **Non-severe** | **Female** | **20** | **15** | **6400** | **64.90** | **<40** |
| **CoV-24** | **Non-severe** | **Male** | **47** | **16** | **204800** | **71.27** | **<40** |
| **CoV-30** | **Non-severe** | **Female** | **50** | **23** | **12800** | **58.54** | **<40** |
| **CoV-34** | **Non-severe** | **Female** | **46** | **16** | **51200** | **72.24** | **<40** |
| **CoV-35** | **Non-severe** | **Female** | **30** | **17** | **12800** | **79.19** | **<40** |
| **CoV-46** | **Non-severe** | **Male** | **41** | **23** | **25600** | **79.38** | **<40** |
| **CoV-47** | **Non-severe** | **Male** | **42** | **25** | **3200** | **90.04** | **47.07** |
| **CoV-51** | **Severe** | **Female** | **34** | **18** | **12800** | **62.08** | **<40** |
| **CoV-54** | **Non-severe** | **Female** | **30** | **18** | **12800** | **35.03** | **<40** |
| **CoV-87** | **Severe** | **Female** | **72** | **22** | **51200** | **21.28** | **<40** |
| **CoV-89** | **Non-severe** | **Female** | **50** | **15** | **6400** | **84.96** | **<40** |
| **CoV-92** | **Severe** | **Female** | **82** | **22** | **102400** | **73.96** | **<40** |
| **CoV-93** | **Non-severe** | **Female** | **47** | **45** | **12800** | **73.66** | **<40** |
