# Supplementary material for: Monoclonal antibodies constructed from COVID-19 convalescent memory B cells exhibit potent binding activity to MERS-CoV spike S2 subunit and other human coronaviruses
Source: Front Immunol. 2022 Dec 22;13:1056272. doi: 10.3389/fimmu.2022.1056272 (PMC9813381; doi:10.3389/fimmu.2022.1056272)
Supplement: Supplementary Figure 3 — Amino acid sequence alignment of the stem region of spike proteins of seven hCoVs. The alignment was performed using spike protein sequences: MERS-CoV (GenBank accession number: AFS88936.1), SARS-CoV-2 (NCBI Reference Sequence: YP_009724390.1), SARS-CoV (NCBI Reference Sequence: NP_828851.1), OC43 (GenBank: AVR40344.1), HKU1 (UniProtKB/Swiss-Prot: Q0ZME7.1), NL63 (GenBank: APF29071.1), and 229E (GenBank: APT69883.1). Sequence alignment was performed using CLUSTALW (Clustal Omega < Multiple Sequence Alignment < EMBL-EBI) and visualized using ESPript 3.0 (ESPript 3.x/ENDscript 2.x). [file Presentation_3.pdf]

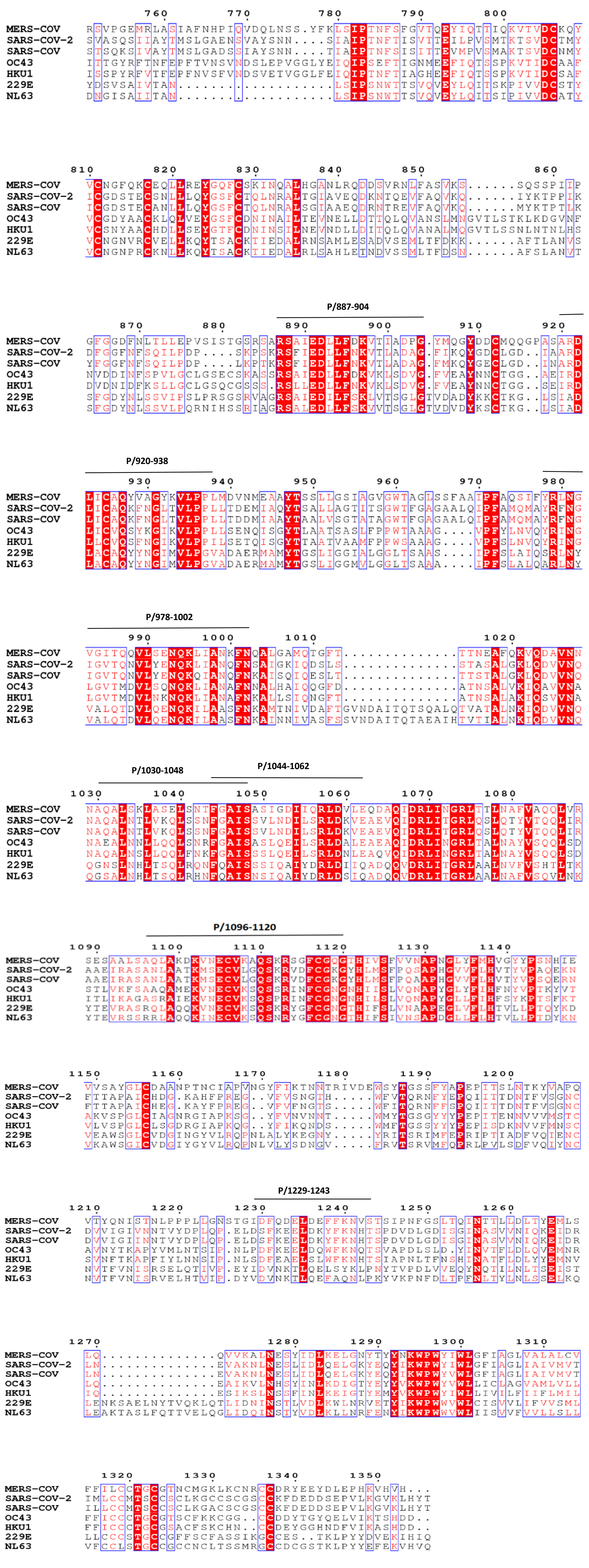
MERS-COV
SARS-COV-2
SARS-COV
OC43
HKU1
229E
NL63
P/887-904
P/920-938
P/978-1002
P/1030-1048
P/1044-1062
P/1096-1120
P/1229-1243
